# Molecular Mapping of Restriction-Site Associated DNA Markers in Allotetraploid Upland Cotton

**DOI:** 10.1371/journal.pone.0124781

**Published:** 2015-04-20

**Authors:** Yangkun Wang, Zhiyuan Ning, Yan Hu, Jiedan Chen, Rui Zhao, Hong Chen, Nijiang Ai, Wangzhen Guo, Tianzhen Zhang

**Affiliations:** 1 National Key Laboratory of Crop Genetics and Germplasm Enhancement, Cotton Hybrid R & D Engineering Center (the Ministry of Education), Nanjing Agricultural University, Nanjing 210095, China; 2 Cotton Research Institute, Xinjiang Academy of Agricultural and Reclamation Sciences, Shihezi 832000, Xinjiang, China; 3 Shihezi Agricultural Sci & Tec Research Center, Shihezi 832000, Xinjiang, China; Nanjing Forestry University, CHINA

## Abstract

Upland cotton (*Gossypium hirsutum* L., 2n = 52, AADD) is an allotetraploid, therefore the discovery of single nucleotide polymorphism (SNP) markers is difficult. The recent emergence of genome complexity reduction technologies based on the next-generation sequencing (NGS) platform has greatly expedited SNP discovery in crops with highly repetitive and complex genomes. Here we applied restriction-site associated DNA (RAD) sequencing technology for *de novo* SNP discovery in allotetraploid cotton. We identified 21,109 SNPs between the two parents and used these for genotyping of 161 recombinant inbred lines (RILs). Finally, a high dense linkage map comprising 4,153 loci over 3500-cM was developed based on the previous result. Using this map quantitative trait locus (QTLs) conferring fiber strength and *Verticillium Wilt* (VW) resistance were mapped to a more accurate region in comparison to the 1576-cM interval determined using the simple sequence repeat (SSR) genetic map. This suggests that the newly constructed map has more power and resolution than the previous SSR map. It will pave the way for the rapid identification of the marker-assisted selection in cotton breeding and cloning of QTL of interest traits.

## Introduction

Upland cotton (*Gossypium hirsutum* L.) accounts for >95% of the world’s cotton production. It is an allotetraploid derived from an allopolyploidization event between *G*. *herbaceum L*. (A_1_) and *G*. *raimondii* (D_5_) Ulbrich that occurred approximately 1–2 million years ago, and contributed the constituent A and D genomes, respectively [1]. With the rapid development of genetic markers, single nucleotide polymorphisms (SNPs) have become the preferred genetic markers of many researchers because of the relatively high cost and limited marker density of more traditional markers.

SNPs are known to be the most abundant genetic markers and are distributed throughout the genome [2–5]. High throughput DNA sequencing technology facilitates the rapid and cost-efficient discovery of large numbers of SNPs. SNP markers provide powerful tools for the construction of genetic and physical maps, marker-assisted selection, map-based cloning, QTL mapping and for other genetic and genomic applications. To date, genome-wide SNP discovery has been applied in a number of crops, such as maize, soybean, rice, oilseed rape. However, in polyploid crops with large and complex genome, successful identification and validation of SNPs is relatively difficult due to the presence of homoeologous loci from the individual subgenomes or paralogous loci from duplicated segments of the genome. Therefore, many complexity reduction approaches such as genotyping-by-sequencing (GBS), IIB digest restriction-site associated DNA (2b-RAD) and reduced-representation libraries (RRLs) based on NGS platforms have been developed [6–7]. These approaches have reduced the complexity of the genome and have been successfully applied in a number of organisms with large complex genomes, including wheat and oilseed rape. One such approach is RAD sequencing. This method relies on cutting DNA with a chosen restriction enzyme, ligating an adapter containing a molecular identifier unique to each sample, and sequencing the captured genomic DNA flanked by restriction enzyme cleavage site using a high throughput sequencing platform to generate RAD-tags. These RAD tags represent a complexity-reduced genome. The alignment of RAD-tags between two parents produces RAD-markers with putative SNP sites and allows the identification of RAD-marker genotypes for all individuals in a mapping population. Furthermore, RAD sequencing can be readily analyzed without a reference genome, which makes the technique particularly applicable to organisms without a complete genome sequence. Therefore, RAD sequencing provides a flexible, inexpensive platform for the genome scale genetic markers mining [8–11]. To our knowledge, this method has not been applied to SNP based association studies in allotetraploid cotton.

In the current study, our aim was to discover, validate and genotype SNP markers in an RIL population. A large dataset of high quality RAD markers was generated. Based on the SNP markers and genotypes of the RILs, we constructed a high-density genetic linkage map. QTLs conferring fiber strength and VW resistance were mapped on the newly constructed map, and further QTLs were tagged. Our results proved RAD sequencing technology was feasible and efficient in QTL mapping using an RIL mapping population in allotetraploid cotton.

## Materials and Methods

### Plant materials and phenotype determination

An RIL population comprising 179 individuals was developed from a single seed descended from a cross between Acala Prema and 86–1, which had previously been used for fiber strength and VW resistance QTL mapping [12]. Fiber strength data from six environments was analyzed by fiber quality investigation came from Ning *et al*.[12], also the result of VW resistance evaluation under field and greenhouse conditions [13]. Field planting has been approved by Nanjing Agric. Univ. No specific permissions were required for these locations/activities since they are pure-line cultivars and the field studies did not involve endangered or protected species.

### DNA extractions

Plant leaf tissue was collected from 6-week-old plants grown in the field. Three young leaves were harvested from each RIL plant and stored at -70°C. Genomic DNA was extracted using the hot CTAB extraction technique as described by Paterson *et al*. [14]. Next, crude DNA extract was purified to remove RNA and protein contaminants using a DNeasy Kit (Qiagen, Valencia, CA). Finally, the purified DNA was suspended in DNase-free water and quantified using Qubit (Invitrogen).

### Construction of the RAD library

Population-specific RAD markers were developed based on the RAD reads from Prema and 86–1 parental DNA, and were genotyped in 161 RIL progeny using the two-enzyme method described by Poland *et al*., with minor modifications [10]. Firstly, high quality genomic DNA (~500ng) was double digested with a combination of restriction endonucleases: *EcoR*I and *Bfa*I [New England Biolabs (NEB), Ipswich, MA]. The mixture was incubated at 37°C for 10 min. The double-digest DNA was then purified with AMPure XP beads (Beckman Coulter Genomics). P1 and P2 adapters, along with 1.0μl T4 DNA ligase (NEB), 4μl 10×T4 DNA ligase buffer, and water, was added to the sample and incubated at room temperature (22°C) for 20 min, before being heat-inactivated for 15 min at 65°C. Thus, adapters containing amplification primer sites, Illumina sequencing primer sites, and a unique barcode were ligated to genomic DNA at the restriction enzyme cut sites. DNA fragments were separated on a 2.0% agarose 0.5× TBE gel and a DNA fraction ranging from 400 to 500 bp in length was isolated using a MinElute Gel Extraction Kit (Qiagen, Valencia, CA). Approximately 20 ng of each isolated sample was amplified by PCR with 25μl Phusion Master Mix (NEB), 5 μl modified Solexa amplification primer, and water to a total volume of 50μl. PCR was carried out according to the Phusion product instructions for a total of 10–12 cycles. The PCR products were cleaned with 1.5× AMPure XP beads, and eluted to 20 μl. 1 μl of the purified PCR product was run on an Agilent Bioanalyzer to quantify its molarity and check the fragment size distribution. Quantified libraries were sequenced on an Illumina Hiseq 2500 platform using the single read (1× 100bp) sequencing module. Sequences are available on the Sequence Read Archive http://www.ncbi.nlm.nih.gov/Traces/sra/, at accession PRJNA273615.

### RAD marker discovery and genotyping

**Fig 1:**
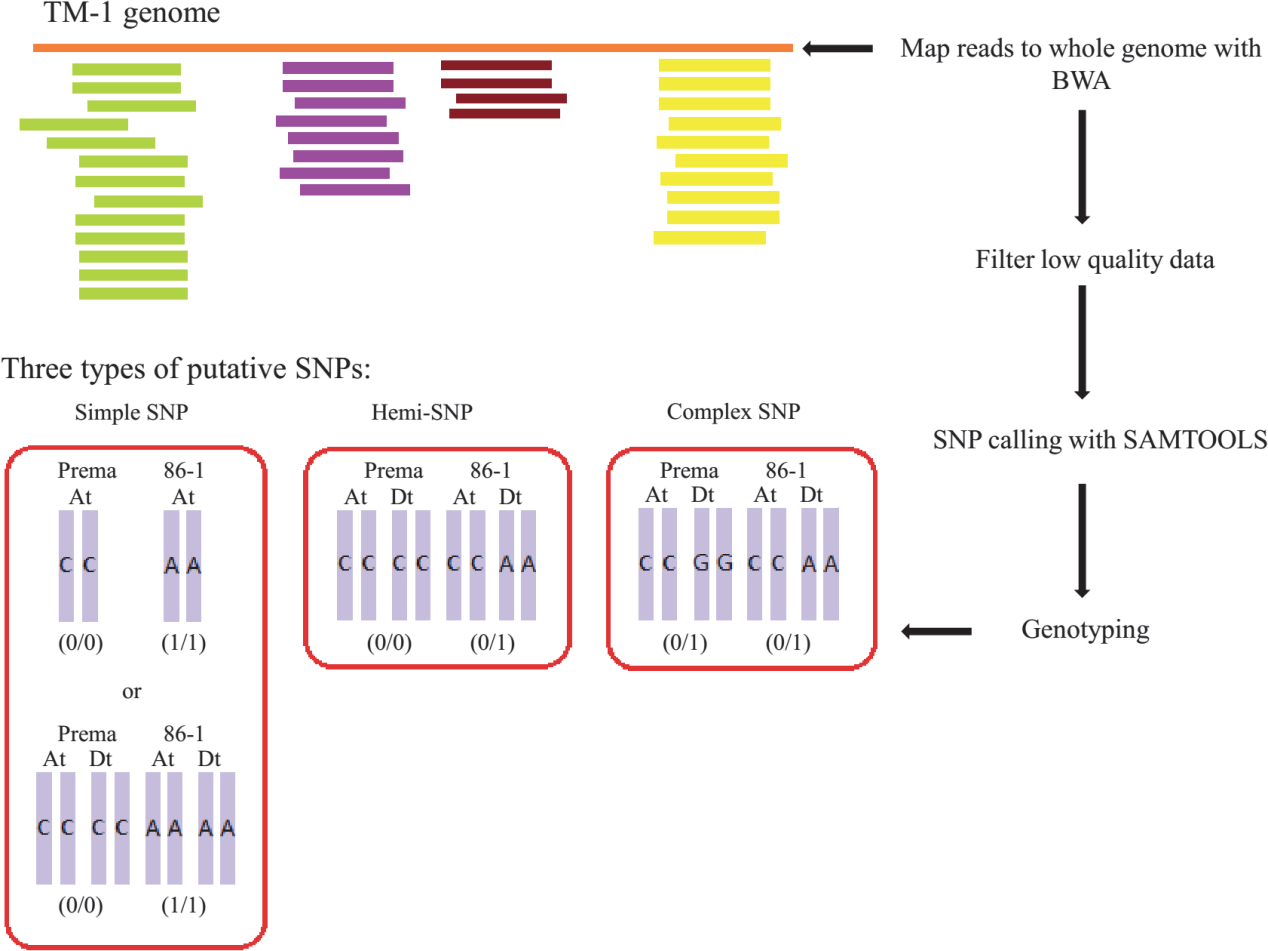
SNP development and genotyping pipeline for cotton RAD markers. Raw reads were aligned to the TM-1 reference genome with BWA software. After filtering and SNP calling, the putative SNPs were divided into three types: simple SNPs with one allele per parent, and complex SNPs which with one allele from one parent and two alleles from the other parent or SNPs with two alleles from both parents.

The SNP discovery was conducted according to pipeline as shown in Fig 1. Raw reads from the parents and RILs were aligned to the tetraploid cotton (TM-1) reference genome (Sequences are available at the Sequence Read Archive http://www.ncbi.nlm.nih.gov/Traces/sra/, at accession PRJNA248163) using BWA software [15]. The reads with a mapping Q-Value <20, and those aligned to multiple-sites in the reference genome, were filtered out. In addition, RAD-tags with a depth ≤10 in parents, and ≤8 in the RIL progeny, were removed due to their lack of coverage for calling SNPs and genotyping. The retained RAD-tags were sent to SAMTOOLS software to be sorted by RAD-loci and were than genotyped in RIL population [16]. Genotyping is more difficult in polyploidy than in diploid species due to multiallele combinations in their genotypes. In theory, SNPs in polyploid species are classified as homoeo-SNPs, simple SNPs, hemi-SNPs or complex SNPs [17–18]. In this study, genotyping was performed using only simple SNPs identified between two parents. Markers were used to construct the genetic map. RAD-markers with a single bi-allelic SNP (called a simple SNP) were screened in the RIL progeny to confirm segregation, while RAD markers with more than 50% missing data in the progeny were excluded.

### Genetic map construction and QTL analysis

Linkage maps were assembled using Joinmap3.0 Version Software with RIL population type code. The input datasets comprised the genotypes of 179 RILs by SSR alleles and 161 RILs by RAD markers. The chromosome locations of the segregation distortion markers were taken from the previously published map [12] and the CMD website. The calculation of the genetic distance between two segregation distortion markers, and the reconstruction of linkage maps were carried out using DistortedMap v1.0 software [19]. Marker groupings were determined using the “Group” command at a maximum recombination frequency threshold of 0.4 and a minimum LOD score greater than 10.0. A ripple was performed after the additional of each locus, and the jump threshold was set to 0.5. The Kosambi mapping function was used to translate recombination frequencies into map distances. Windows QTL Cartographer 2.5 was used to identify QTLs by composite interval mapping (CIM) [20–21]. Quantitative trait loci were determined to be significant if the corresponding likelihood ratio score was greater than 11.5 (equal to the LOD score of 2.5). The percentage of the phenotypic variance (PV) explained by a QTL was estimated at the highest probability peaks. The graphic representation of the linkage group was created by MapChart2.2 [22].

## Results

### RAD marker discovery and genotyping

Massively parallel sequencing of RAD libraries generated about 20 million and 14 million RAD-tags 100bp in length in Prema and 86–1, respectively. Altogether, about 2,294 million RAD-tags were obtained from 169 RIL progeny. On average, the number of reads for each RIL progeny was 13,575,837; ranging from 53,666 to 44,299,744. Eight progeny with <2,000,000 reads were excluded from the mapping dataset, leaving 161 progeny for genotyping (Table 1). After filtering the homoeo-SNPs, a total of 21,109 intraspecific SNPs were identified between the two parents and were classified as three types (Table 2). Of all intraspecific SNPs, 14,445 (68.43%) were simple SNPs. These simple SNPs were used to genotype the 161 RILs. After genotyping, 5,771 RAD-markers containing 6,851 simple SNPs (1~5 SNPs per RAD-marker) were identified in the 161 progeny (S1 Table). A Chi squared goodness-of-fit test showed that about one-third of the loci fitted the segregation ratio 1:1, and two-thirds of the loci had distorted segregation ratios. This was confirmed by an intraspecific genetic map containing 279 SSR markers from this population [12].

**Table 1:** RAD-seq statistics summary of two-enzyme library from parents and their RIL progeny.

Statistic	Number
**Total Illumina reads**	2,647,620,011
**Total reads containing barcode and restriction**	2,515,239,010
**Reads per progeny containing restriction overhang (1 mismatch allowed) (range)**	53,666–44,299,744
**Mean of reads per progeny**	13,575,837
**SNPs between parents**	21,109
**SNPs were identified in the RIL progeny**	6,851
**RAD markers from which SNPs were derived**	5,771

**Table 2:** Identified putative SNPs classified by SNP type.

SNP Type	Desciption	Prema	86–1	Identified SNPs	Persentage(%)
**simple SNP**	SNPs with one allele per parent	C	A	14,445	68.43
**hemi-SNP**	SNPs with one allele in one parent and two alleles in the other	C	C/A	6340	30.03
**complex SNP**	SNPs with two alleles per parent	C/G	C/A	324	1.53

### Genetic map construction

The genetic map was constructed using 5,771 RAD-markers and 304 SSR markers for which genotypic data were available [12]. This gave rise to a genetic map consisting of 103 linkage groups representing the 26 chromosomes in allotetraploid cotton. The map, spanning a total of 3499.69cM, comprises 3,984 RAD markers and 169 SSR markers (S1 File, S2 Table and Table 3). This map was aligned with a previously published genetic map using common SSR markers [12]. Because the LOD score was increased to 10.0 in order to reduce the interference of the HSV that could not distinguish after unique alignment, only half of the SSR markers were included in this new genetic map, compared with the old SSR genetic map, which contained 279 SSR markers.

**Table 3:** The information of chromosome in the genetic map based on SNPs of tetraploid cotton.

Chr.	No. RAD-Tags	groups	SSRs	SNPs
**A1(Chr.1)**	261	4	10	319
**A2(Chr.2)**	80	2	5	93
**A3(Chr.3)**	68	3	9	74
**A4(Chr.4)**	105	3	3	111
**A5(Chr.5)**	144	3	9	168
**A6(Chr.6)**	53	3	4	59
**A7(Chr.7)**	298	7	9	356
**A8(Chr.8)**	218	2	4	263
**A9(Chr.9)**	199	4	4	242
**A10(Chr.10)**	141	3	6	171
**A11(Chr.11)**	294	6	11	330
**A12(Chr.12)**	119	5	3	131
**A13(Chr.13)**	145	5	3	165
**At-Total**	2125	50	80	2482
**D1(Chr.15)**	246	4	8	300
**D2(Chr.14)**	208	3	11	259
**D3(Chr.17)**	100	2	7	116
**D4(Chr.22)**	84	3	9	98
**D5(Chr.19)**	123	3	10	143
**D6(Chr.25)**	151	2	9	182
**D7(Chr.16)**	166	5	5	206
**D8(Chr.24)**	303	5	8	355
**D9(Chr.23)**	87	8	3	111
**D10(Chr.20)**	205	7	11	253
**D11(Chr.21)**	64	3	4	77
**D12(Chr.26)**	67	3	2	76
**D13(Chr.18)**	55	5	2	59
**Dt-Total**	1859	53	89	2235
**Total**	3984	103	169	4717

Our data also suggested that the RIL progeny derived from 86–1 and Prema showed segregation distortion; with 3,680 (63.77%) distorted markers out of the 5,771 markers scored. Similarly, in the F_2:3_ (MD5678ne × Prema) population, 36 of 113 RFLP loci deviated from the expected 3:1 ratio for a dominant locus and the 1:2:1 ratio for a co-dominant locus, and 11 loci that exhibited distortion (χ2 > 25.0) showed a higher than expected allelic frequency from the Prema parent. This, suggests that there is an allelic preference for this parent in this population [23]. It is clear that segregation distortion is a feature of most intercrosses between introgression lines and G. *hirsutum* L. [14, 26, 40, 41, 42].

### QTL mapping using an enhanced RAD genetic map

**Fig 2:**
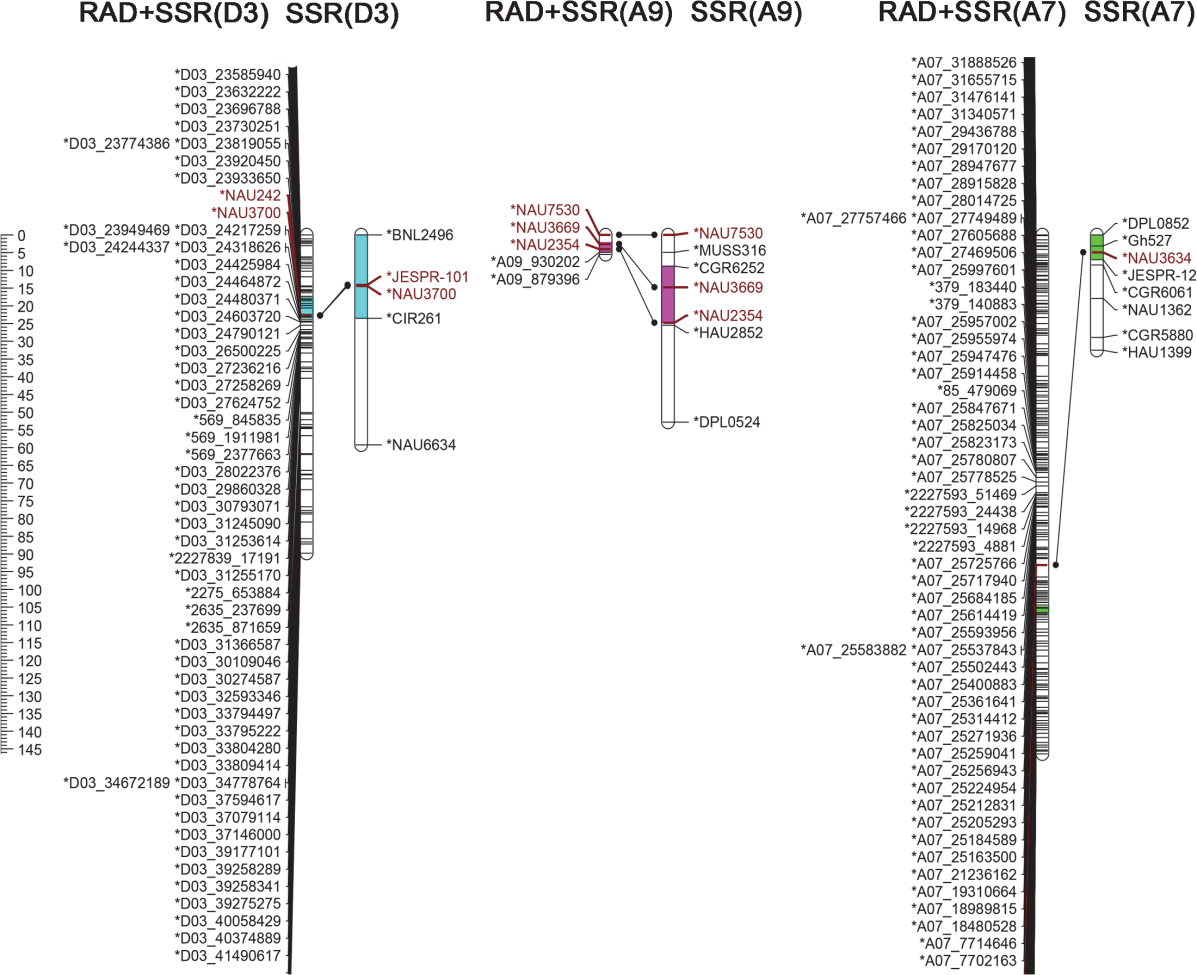
QTLs for fiber strength in two maps. Three QTLs for fiber strength, *qFS-D3-1*, *qFS-A9-1*, *qFS-A7-1* were identified on both maps, with the different colors indicating different 2-LOD intervals for each QTL. Maps were constructed with RAD markers plus SSR markers, and with SSR markers alone. Lines connecting the same markers in these two maps correlated the trait: these markers are colored brown.

**Fig 3:**
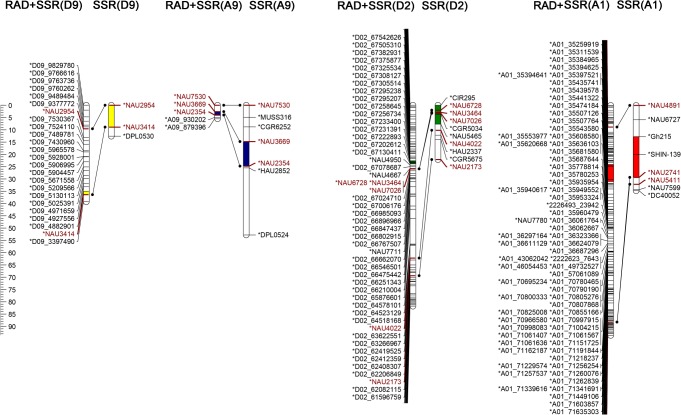
QTLs for VW resistance in two maps. Four QTLs for VW resistance, *qVW-D9-1*, *qVW-A9-1*, *qVW-D2-1*, *qVW-A1-1* were identified on both maps, with different colors indicating different 2-LOD intervals for each QTL. Maps were constructed with RAD markers plus SSR markers, and with SSR markers alone. Lines connecting the same markers in these two maps correlated the trait: these markers are colored brown.

The newly constructed enhanced RAD genetic map was used to tag QTLs for fiber strength and VW resistance. Thirteen non-redundant QTLs for fiber strength and ten non-redundant QTLs for VW resistance were detected (Table 4). Most of these QTLs were also detected by Ning *et al*. using the SSR genetic map, including *qFS-D3-1*, *qFS-A9-1*, and *qFS-A7-1* for fiber strength, and *qVW-D9-1*, *qVW-A9-1*, *qVW-D2-1*, and *qVW-A1-1* for VW resistance (Figs 2 and 3).

**Table 4:** QTLs fiber strength and VW-resistance detected by CIM in the RILs in different environments.

Trait	QTL	Chromosome	Position(cM)	LOD	Flanking marker	Additive_effect	R²(%)	Origin	Enviroments
**Fiber strength**	qVW-A5-1	A5-1	16.71	3.016287	A05_28741092 and A05_28119729	1.0102	1.649317	Prema	Akasu,2010
**Fiber strength**	qVW-A5-1	A5-1	14.21	4.60152	A05_28472100 and A05_28551071	1.5771	6.265382	Prema	Dafeng,2010
**Fiber strength**	qVW-A5-1	A5-1	10.71	4.89468	NAU2140 and A05_28347173	1.083	2.358131	Prema	Shihezi,2009
**Fiber strength**	qFS-A7-1	A7-3	41.21	5.092291	A07_5985512 and A07_6000491	2.2982	9.523675	Prema	Shihezi,2011
**Fiber strength**	qFS-A7-1	A7-3	41.01	4.445168	A07_5985512 and A07_5986659	1.1211	2.413734	Prema	Shihezi,2009
**Fiber strength**	qFS-A7-1	A7-3	41.21	3.62215	A07_5985512 and A07_5986659	1.1407	4.304667	Prema	Jiangpu,2009
**Fiber strength**	qFS-A9-1	A9-4	2.61	8.462541	NAU3669 and NAU2354	2.4398	9.372284	Prema	Shihezi,2011
**Fiber strength**	qFS-A9-1	A9-4	2.61	9.090119	NAU3669 and NAU2354	1.0471	1.521089	Prema	Akasu,2010
**Fiber strength**	qFS-A9-1	A9-4	2.61	4.870793	NAU3669 and A09_930202	1.2817	6.207383	Prema	Dafeng,2010
**Fiber strength**	qFS-D3-1	D3-1	66.71	5.463626	D03_24318626 and D03_23920450	4.349	14.70778	Prema	Shihezi,2011
**Fiber strength**	qFS-D3-1	D3-1	67.21	14.58415	D03_23949469 and D03_23933650	1.875	2.112363	Prema	Akasu,2010
**Fiber strength**	qFS-D3-1	D3-1	71.21	7.211726	D03_23730251 and D03_23585940	1.6921	4.721868	Prema	Sanya,2009
**Fiber strength**	qFS-D3-1	D3-1	67.21	11.23561	D03_23949469 and D03_23933650	2.242	5.233972	Prema	Jiangpu,2009
**VW resistance**	qVW-A1-1	A1-3	24.61	3.986971	A01_22210881 and 1723_15192	-7.7866	24.27434	Prema	vd8 in greenhouse
**VW resistance**	qVW-A1-1	A1-3	25.81	3.107492	A01_22798197 and 1723_114141	-13.1188	12.4457	Prema	Shihezi,2010
**VW resistance**	qVW-D2-1	D2-3	58.51	3.144408	D02_67130411 and D02_67233400	-11.2664	8.00717	Prema	Shihezi,2011
**VW resistance**	qVW-A6-1	A6-1	3.71	6.414767	NAU6973 and A06_2845454	10.5553	44.60596	86–1	vd8 in greenhouse
**VW resistance**	qVW-A9-1	A9-4	2.61	7.611292	NAU3669 and NAU2354	-8.8356	31.25531	Prema	vd8 in greenhouse
**VW resistance**	qVW-D9-1	D9-1	3.91	4.473398	NAU3414 and D09_4882901	-6.2136	15.45747	Prema	vd8 in greenhouse
**VW resistance**	qVW-D9-1	D9-1	3.91	23.53095	NAU3414 and D09_4882901	-27.8261	55.99325	Prema	Shihezi,2010
**VW resistance**	qVW-D9-1	D9-1	3.91	32.59718	NAU3414 and D09_4882901	-35.5163	79.57271	Prema	Shihezi,2011
**VW resistance**	qVW-A11-1	A11-3	23.51	6.29316	A11_77497859 and A11_76565350	-21.2239	28.41575	Prema	Shihezi,2011

The QTL *qFS-D3-1*, derived from the parent Prema, was located in D3, which was simultaneously detected in four environments. This QTL explained 2.11%~14.71% of PV, with LOD scores ranging from 5.46 to 14.58. It was localized to an area between two RAD-markers, D03_23585940 and D03_24318626, with 2-LOD intervals <6 cM (Table 4). The SSR marker NAU3700, which was also located between these two RAD-markers, has previously been mapped to D3 [12, 27]. QTL *qFS-D3-1* was located between 23,585,940 and 24,217,259 bp on D3, with the most significant marker, NAU3700, placing it at 23,942,014 bp. Additionally, the QTLs *qFS-A9-1* and *qFS-A7-1* were simultaneously detected in three environments, and explained 1.52%-9.37% of PV and 2.42%-9.52% of PV, with LOD scores ranging from 4.87 to 9.09, and 3.62 to 5.09, respectively. These two QTLs were also detected by Ning *et al*. in three environments and combined analysis [12].

The QTL *qVW-D9-1*, the major VW resistant QTL originating from Prema and located in D9, was detected in three environments. This QTL explained 15.46%~79.57% of PV, with LOD scores ranging from 4.447 to 32.60. This resistant QTL was flanked by SSR marker NAU3414 and RAD-marker D09_4882901, with 2-LOD intervals ≤2 cM. This QTL was also detected by Jiang *et al*. [28], who used an intraspecific population of 229 F2 individuals derived from a cross between two Upland cotton cultivars, Prema (VW-resistant) and Junmian 1 (susceptible). Furthermore, this QTL was also detected by Ning *et al*. [12] using SSR markers in the same population. Ning *et al*. found the genetic distance between the flanking markers to be 8.9 cM, while our study showed a much smaller distance 1.5 cM between the flanking markers, NAU3414 and D09_4882901. The candidate genes identified by this QTL interval contained several disease resistance-responsive (dirigent-like protein) protein family-related genes. This suggests that mapping with RAD markers to identify QTLs is more efficient than with other molecular markers. In addition to this major QTL *qVW-D9-1*, QTLs *qVW-A9-1*, *qVW-D2-1*, *qVW-A1-1* with LOD scores ranging from 3.11 to 7.61, were detected on the enhanced RAD genetic map, as well as by Ning *et al*. [13]. These QTLs explained 8.01%~31.25% of PV. The distance between the flanking markers of each QTL on the new map was much samller than the distance on the SSR genetic map.

We also tagged more QTLs for these two traits compared with the result by using the same RIL population, these QTLs included *qVW-A6-1*, *qVW-A11-1*, and *qVW-A13-1* for VW resistance, and *qFS-A5-1* for fiber strength, which was detected in three environments. The QTLs for VW resistance, with significant additive effects, explained 21.20%~44.6% of PV, and might be newly identified QTLs for this trait. The QTL *qFS-A5-1*, which explained 1.64%~6.327% of PV with LOD score ranging from 3.02 to 4.89, was also detected by Lacape *et al*. [29] in an inter-specific G. barbadense × G. hirsutum RIL population for fiber characteristics in 11 independent experiments under field and glasshouse conditions. These results may help elucidate the genetic basis and distribution of valuable QTLs in the cotton genome and facilitate the application of marker-assisted selection to improve VW resistance and fiber quality.

## Discussion

### Development of markers in genetic study

In order to detect the allelic variation within different samples at the DNA level, researchers have developed a variety of molecular markers such as RFLP, RAPD, SSRs, InDels and SNPs. SNP markers proved to be universal as well as the most abundant forms of genetic variation among individuals of the same species. In particular, SNPs have become increasingly popular in the field of plant molecular genetics due to the drastic decrease in the cost of high throughput sequencing.

SNP markers have been widely used in genetic studies, mainly for the purposes of building genetic maps, investigating population genomics, and phylogeography. For instance, Poland *et al*. were able to map over 34,000 SNPs and 240,000 tags on the Oregon Wolfe Barley reference map, and 20,000 SNPs and 367,000 tags on the Synthetic W97846Opata85 (SynOpDH) wheat reference map, they also constructed a *de novo* genetic map using only SNP markers from GBS data [10]. Emerson *et al*. identified 3,714 SNPs by RAD sequencing, revealed previously unresolved genetic structure and direction of evolution in the pitcher plant mosquito, *Wyeomyia smithii*, which from a southern Appalachian Mountain refugium following recession of the Laurentide Ice Sheet at 22,000–19,000 B.P. [24]. Despite its advantages, studies on genome-wide SNP discovery in allotetraploid cotton are sparse. To date, in cotton, the identification of SNPs has largely been identified based on short genomic sequence or expressed sequence tag (EST) data [30–32]. Traditional methods for SNP discovery are time-consuming and expensive. However, large, complex and nature polyploidy of its genome hampers large scale SNP mining in cotton. Therefore, it is important to employ genome complexity reduction techniques coupled with NGS technologies for genome-wide SNP discovery in cotton. In our study, we successfully identified a large number of SNP markers based on a genome complexity reduction technology called RAD, and simultaneously genotyped many individuals. This method reduces the complexity of the genome and detect sequence variations in coding and non-coding regions across the genome; these advantages have led to the wide use of RAD sequencing in QTL mapping and fine mapping [33–35]. Our results demonstrate that SNP discovery based on RAD sequencing is efficient in the intra-specific *G*. *hirsutum* populations. Moreover, these SNPs can subsequently serve as a resource for a SNP chips in the development of large genotyping arrays. The RAD sequencing approach has the flexibility to assay different numbers of markers depending on the choice of restriction enzyme. It is clear that as the depth of sequencing was increased, more polymorphisms were detected. However, obtaining a high sequencing depth might not be economically efficient; therefore, restriction enzymes and sequencing depth should be chosen based on the expected degree of polymorphism.

### SNP validation in polyploidy crops

Validation of SNPs in polyploidy crops is difficult due to the presence of HSVs, PSVs, sequence errors, as well as the true SNPs [36–38]. The software STACKS [39] was developed to analyze data from RAD sequencing with or without a reference genome [24–25]. However, as STACKS was developed for diploid analysis, it could not be used in our study. Therefore, the software, SAMTOOLS, was utilized for SNP calling instead. The putative intraspecific SNPs between parents were classified into three types: simple SNP which can be treated as diploid SNPs during the analysis, contain several HSVs that can not be distinguished after unique alignment. To ensure the authenticity of the genetic map based on these SNPs, the minimum LOD score was increased to 10.0 to reduce the influence of these HSVs and other two types of ‘complex’ SNP: those with one allele from one parent and two alleles from the other parent (hemi-SNPs), and SNPs with two alleles from both parents. These complex SNPs were excluded due to the large quantity of missing data in the progeny. This screening method may reduce the number of SNPs compared with the STACKS method.

### QTL reassessment by using the enhanced RAD genetic map

Our reassessment of QTLs for fiber strength and VW resistance by applying RAD sequencing to identify SNPs indicates that this approach is applicable to polyploidy plants. The genetic components of fiber strength and VW resistance in upland cotton were also reexamined in this study. However, it remains unclear which genes play important roles in fiber strength determination; subsequent fine mapping of the F2 population by through several different intercrosses between 86–1 and RILs with high fiber strength will contribute to our knowledge on this topic. The VW resistance of cotton is likely to be genetically determined by a major resistance QTL, but it is also affected by environment conditions. Further study into the mechanism of expression of this trait is needed.

## Supporting Information

S1 File Linkage map of Acala Prema × 86–1 population using restriction-associated DNA (RAD) and SSR marker. (PDF)

S1 Table RAD marker information and genotypes. (XLSX)

S2 Table Genetic map information. (XLSX)
