# Supplementary material for: Molecular Mapping of Restriction-Site Associated DNA Markers in Allotetraploid Upland Cotton
Source: PLoS One. 2015 Apr 20;10(4):e0124781. doi: 10.1371/journal.pone.0124781 (PMC4403916; doi:10.1371/journal.pone.0124781)
Supplement: S1 File — (PDF) [file pone.0124781.s001.pdf]

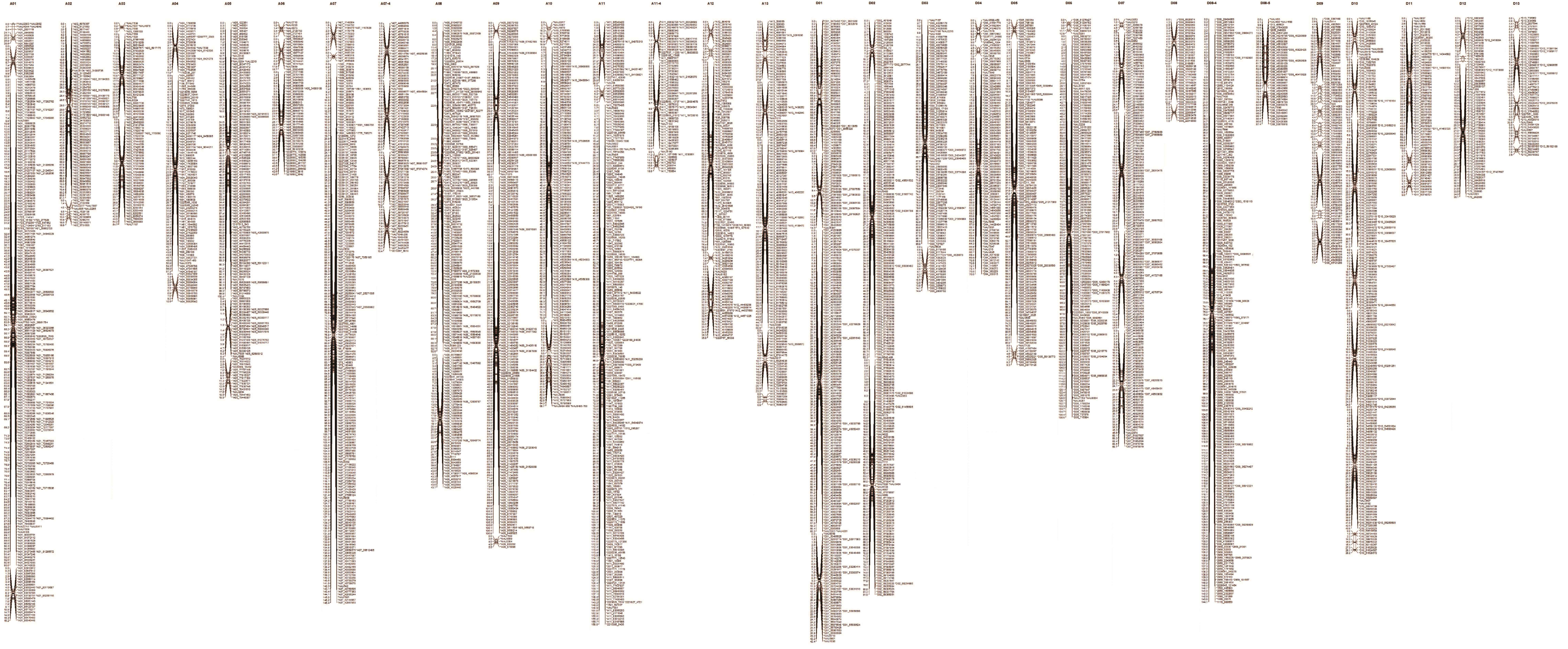
A01
A02
A03
A04
A05
A06
A07
A07-4
A08
A09
A10
A11
A11-4
A12
A13
D01
D02
D03
D04
D05
D06
D07
D08
D08-4
D08-5
D09
D10
D11
D12
D13
